# The Economics of Dementia-Care Mapping in Nursing Homes: A Cluster-Randomised Controlled Trial

**DOI:** 10.1371/journal.pone.0086662

**Published:** 2014-01-28

**Authors:** Geertje van de Ven, Irena Draskovic, Elke van Herpen, Raymond T. C. M. Koopmans, Rogier Donders, Sytse U. Zuidema, Eddy M. M. Adang, Myrra J. F. J. Vernooij-Dassen

**Affiliations:** 1 Department of Primary and Community Care, Radboud University Nijmegen Medical Centre, Nijmegen, Gelderland, The Netherlands; 2 Department for Health Evidence, Radboud University Nijmegen Medical Centre, Nijmegen, Gelderland, The Netherlands; 3 Scientific Institute for Quality of Healthcare, Radboud University Nijmegen Medical Centre, Nijmegen, Gelderland, The Netherlands; 4 Department of General Practice, University of Groningen, University Medical Centre Groningen, Groningen, Groningen, The Netherlands; 5 Kalorama Foundation, Beek-Ubbergen, Gelderland, The Netherlands; 6 Radboud Alzheimer Centre, Radboud University Nijmegen Medical Centre, Nijmegen, Gelderland, The Netherlands; University of Glasgow, United Kingdom

## Abstract

**Background:**

Dementia-care mapping (DCM) is a cyclic intervention aiming at reducing neuropsychiatric symptoms in people with dementia in nursing homes. Alongside an 18-month cluster-randomized controlled trial in which we studied the effectiveness of DCM on residents and staff outcomes, we investigated differences in costs of care between DCM and usual care in nursing homes.

**Methods:**

Dementia special care units were randomly assigned to DCM or usual care. Nurses from the intervention care homes received DCM training, a DCM organizational briefing day and conducted the 4-months DCM-intervention twice during the study. A single DCM cycle consists of observation, feedback to the staff, and action plans for the residents. We measured costs related to health care consumption, falls and psychotropic drug use at the resident level and absenteeism at the staff level. Data were extracted from resident files and the nursing home records. Prizes were determined using the Dutch manual of health care cost and the cost prices delivered by a pharmacy and a nursing home. Total costs were evaluated by means of linear mixed-effect models for longitudinal data, with the unit as a random effect to correct for dependencies within units.

**Results:**

34 units from 11 nursing homes, including 318 residents and 376 nursing staff members participated in the cost analyses. Analyses showed no difference in total costs. However certain changes within costs could be noticed. The intervention group showed lower costs associated with outpatient hospital appointments over time (p = 0^.^05) than the control group. In both groups, the number of falls, costs associated with the elderly-care physician and nurse practitioner increased equally during the study (p<0^.^02).

**Conclusions:**

DCM is a cost-neutral intervention. It effectively reduces outpatient hospital appointments compared to usual care. Other considerations than costs, such as nursing homes’ preferences, may determine whether they adopt the DCM method.

**Trial Registration:**

Dutch Trials Registry NTR2314

## Introduction

Care for the elderly with dementia is expensive. In 2005, 4.7% of the total health care costs in the Netherlands were spend on dementia, which is US $425.000.000 [1]. Healthcare costs associated with dementia are predicted to rise with the increasing prevalence [2]. The most prevalent resident and staff problem in nursing home dementia care is neuropsychiatric symptoms (NPSs), which 80–90% of the nursing home residents with dementia have [3]. The high prevalence of NPSs is associated with increased demands on staff resources, job-related stress, burnout, and staff turnover [4]. Managing the high health care expenditures related to NPSs, without compromising the quality of care is not a trivial task.

Evidence suggests that different types of person-centered care (PCC) may reduce NPSs and improve both resident and staff outcomes [5]–[7]. There are examples of PCC interventions for nursing home residents with dementia that have been shown to lower the rate of NPSs, falls, and the use of psychotropic drugs [8], [9]. Dementia-care mapping (DCM) is a person-centred, multicomponent intervention developed by the Bradford Dementia Group at the University of Bradford in the UK and is based on Kitwood’s social-psychological theory of personhood in dementia [10]. This theory states that much of the ill-being that people with dementia experience is due to negative environmental influences, including staff attitudes and care practices. DCM is a cyclic intervention consisting of three components: systematic observation, feedback to the staff, and action plans. The action plans are developed by the nursing staff and are based on the observation of the actual needs of the residents. This method allows for timely initiation of tailor-made interventions at the individual level (residents and caregivers) and the group level (nursing teams and multi-disciplinary teams), as well as at the levels of management and organization. In short, DCM is a multi-component intervention aiming at synergistically implementing diverse single-scope interventions to sustainably improve the quality and effectiveness of care [11].

We started a cluster-randomized controlled trial evaluating the effectiveness of DCM in Dutch nursing homes in 2010. The design and the results of this trial on resident and staff outcomes are published earlier [12], [13]. Because of the importance of economic considerations in the implementation of new interventions, we also performed a cost analysis. Since we found no effect in our trial on our primary outcome of agitation, we used a cost minimization analysis to investigate the differences in costs of care.

## Methods

### Participants

The supporting CONSORT checklist, the protocol for this trial and the research proposal are available as supporting information; see Checklist S1, Protocol S1 and Research Proposal S1.The design of the trial has been published previously [13]. We recruited nursing homes by sending invitational letters and approaching nursing homes that already had contact with DCM Netherlands. Care for people with dementia in the Netherlands is generally provided in dementia special care units. Staff in Dutch nursing homes includes nurses, elderly-care physicians, physical therapists, occupational therapists, dietitians, and psychologists, all of whom are employed by the nursing home. Staff in Dutch nursing homes receive a fixed salary based on the number of hours they work, independent of the services they provide [14], [15]. The study sample consisted of residents with dementia and their formal caregivers. Inclusion criteria for the residents required a diagnosis of dementia established by elderly-care physicians according to the dementia criteria of the *Diagnostic and statistical manual of mental disorders IV*, [16] approval of the elderly-care physicians for inclusion, age of 65 years or more, at least 1 neuropsychiatric symptom in the last 2 weeks as assessed with the Neuropsychiatric Inventory – Nursing Home, informed consent of the resident or his/her family, and the ability of the resident to use the common areas, such as the shared living room, for at least 4 hours a day. Residents with an estimated life expectancy of 6 weeks or less and those who were physically unable to spend time in common areas of the unit were not included in the study. Participants lost to follow-up were replaced by new participants throughout the study. The trial is registered with the Dutch Trials Registry, number NTR2314 (http://www.trialregister.nl/trialreg/admin/rctview.asp?TC=2314).

### Ethical Statement

Written informed consent was obtained from the family of the residents. In those cases in which the resident signed the informed consent form, also the family or legal representative provided a signature for consent. The Committee on Research Involving Human Subjects in the Arnhem-Nijmegen Region approved the study participation.

### DCM-Intervention

The managers at the intervention nursing homes selected staff members who were interested in becoming certified DCM-mappers and who met the competency requirements set by DCM Netherlands. A total of 10 staff members, 2 from each intervention nursing home, attended the DCM basic and advanced training provided by DCM Netherlands and became certified DCM-mappers. An advanced level certification means that the mapper is qualified to conduct and report structured DCM observations, provide feedback to the staff, and instruct and support them in drawing up action plans for the residents. At the end of the DCM training, a member of DCM Netherlands and a researcher (AP and GV) provided a DCM organizational briefing day for the intervention nursing homes. After completing the training and the organizational briefing day, the trained mappers had to complete at least 2 DCM cycles. A single DCM cycle consists of observation, feedback, and action plans. The control group residents received usual care during the trial. The control nursing homes were offered the DCM training, to take place after the trial. The study period started in October 2010 and lasted until April 2012.

### Costs of the DCM-Intervention

For the purpose of calculating the costs of the DCM intervention, we included the following activities: DCM basic and advanced training, mapping exercise, inter-rater reliability test, observation, preparing the DCM reports, and feedback sessions.

Ten staff members (2 from each intervention nursing home) attended the DCM basic training (US $979.99 per attendee) and the DCM advanced training (US $1371.98 per attendee) provided by DCM Netherlands. We also included the nursing staff hourly wages (32 hours for the basic training and 32 hours for the advanced training). Additionally, we included the hourly wages of all the hours spend on DCM by the mappers. Every mapper did a mapping exercise (6 hours) and an inter-rater reliability test (1.5 hours). The actual hours spent in observation were extracted from the raw data sheets in the DCM reports. The feedback sessions (2 hours each) and the preparation of DCM reports (8 hours each) were standardized. The costs of the hourly wages were covered by a representative nursing home (US $27.68). We used the exchange rate of EUR 1.00 = US $1.318.

We calculated the implementation costs per unit based on the invested hours in implementation activities during the trial. To calculate the mean unit costs per resident per day, we divided the total costs of implementing the DCM intervention by the number of residents in the unit and the days of the study period (549). The mean unit costs per resident per day were taken into account for a baseline period of 6 months (T0), 6 months following the first DCM cycle (T1) and 6 months following the second DCM cycle (T2).

### Outcome Measures

We analyzed the costs from a health care perspective. We used the following outcome measures, based on the aim of DCM to reduce these: health care consumption, number of falls, and psychotropic drug use at the resident level; and absenteeism at the staff level. Data for the economic analysis were collected over a period of 18 months, divided into three periods of 6 months: T0, T1 and T2.

A research assistant and/or a researcher (FB, EH, and GV) visited all nursing homes at the end of the trial to obtain information about all outcome measures. The number of contacts with the nursing home’s health care professionals (elderly-care physician, nurse practitioner, psychologist, social worker, occupational therapist, and dietitian) and the hospitals were extracted from the resident files. The calculation of costs for these contacts was based on a manual for health care cost analysis [17], and the hourly wages of the nursing home’s health care professionals were covered by a nursing home. The number of falls was obtained from the nursing home records at the unit level. While the costs of falls are included in the other outcome measures, such as outpatient hospital appointments, we only present the frequency of falls. Information about the residents’ psychotropic drug use (antipsychotics, antidepressants, hypnotics, anxiolytics, anticonvulsants, and antidementia drugs) was collected at three times, set in the middle of each study period. Data about the use of all psychotropic drugs were collected and detailed to the drug, the dosage, and the regularity of use. Psychotropic drug prescriptions for incidental use were discarded. The pharmacy of the Medical Center of the Radboud University of Nijmegen provided the prices for the products. We used the pharmaceutical prices of generic products, since the DCM intervention is not likely to affect the choice of generic products or brand names. Outcome measures were calculated for each study period per resident, per day.

Data about staff absenteeism was collected at the unit level from the nursing home record system. The costs of absenteeism were based on the hourly wages of the nursing staff, and were provided by a nursing home.

Table 1 details the key unit costs, together with their sources. The baseline characteristics of residents were extracted from the available resident files, whereas staff baseline characteristics were acquired from a survey.

**Table 1:** Key Unit Costs in U.S. Dollars Used to Value Resource Use Measured in the Trial (2010–2012).

	Costs in Dollars	Source of Costs
Hospital		
Outpatient clinic		
University hospital	170.01/contact	1
Regular hospital	84.35/contact	1
Unknown hospital	94.89/contact	1
Inpatient		
University hospital	757.80/day	1
Regular hospital	573.29/day	1
Unknown hospital	734.07/day	1
Emergency department	199/contact	1
Ambulance	436.23/ride	1
Drugs		
Psychotropic drugs	Various	2
Nursing home’s health care professionals		
Elderly-care physician	47.08/contact	3
Nurse practitioner	25.70/contact	3
Psychologist	77.11/contact	3
Social worker	32.76/contact	3
Physical therapist	28.73/contact	3
Occupational therapist	28.73/contact	3
Dietitian	26.25/contact	3
Nursing staff	27.68/hour	3

Sources:

1. Hakkaart-van Oijen et al. 2010.

2. Unit costs at Radboud University Hospital 2012.

3. Professionals contacted for an indication of gross costs in 2012.

### Statistical Analysis

Analyses were based on the principle of intention to treat; all data were analyzed in their randomized condition. The analyses included all randomized and newly included residents and staff members, of whom we had information for at least 1 period. We used the following outcome measures: health care consumption, number of falls, and psychotropic drug use at the resident level; and absenteeism at the staff level. The effects of DCM on costs were evaluated by means of linear mixed-effect models for longitudinal data, with the unit as a random effect to correct for dependencies within units. We did not account for dependencies within nursing homes, because not all nursing homes participated in the study with more than one unit. The control variables used in the studywise minimization [18] were treated as covariates: the size of the nursing home, number of residents per unit, and ratio of formal caregivers to residents. We assumed an AR1 correlation structure with heterogeneous covariance for the residuals to correct for dependencies caused by repeated measurements. The effects estimated for the outcome variables were the main effect of the groups (intervention and control), the main effect of time (T0, T1, and T2), and the interaction between group and time. The DCM implementation costs were included in the total costs. Healthcare consumption and psychotropic drug use were analyzed at the resident level, whereas falls, absenteeism, total resident-based costs (healthcare consumption and drug use) and total costs (health care consumption and drug use, absenteeism, and intervention costs) were analyzed at the unit level. Outcomes analyzed at the unit level were corrected for the numbers of residents and staff members per unit. Two-sided values of p<0·05 were deemed statistically significant. Statistical analyses were carried out with SPSS version 18 (SPSS, Chicago, Ill.).

## Results

### Trial Participants

**Figure 1:**
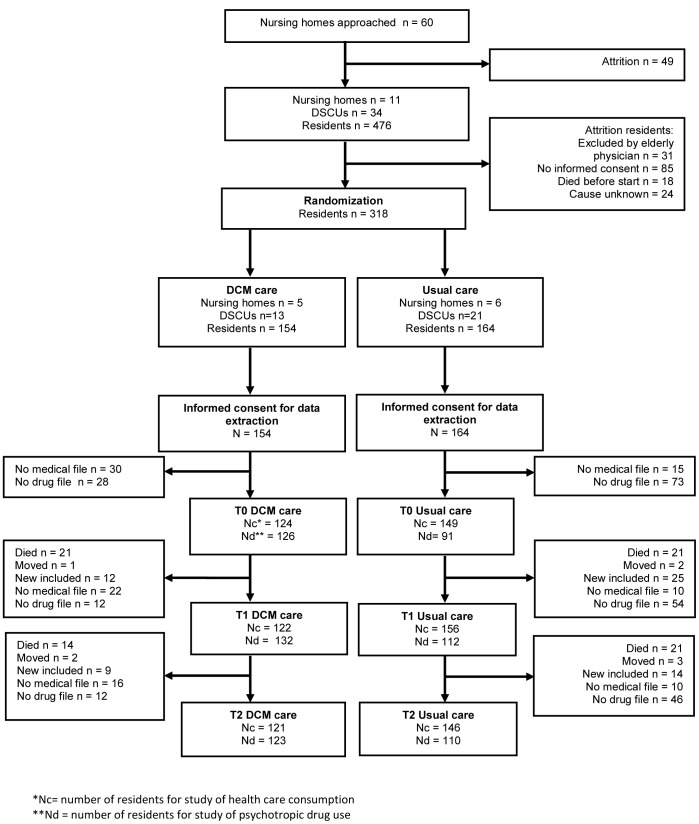
Flow chart of nursing homes and residents.

Thirty-four units from 11 nursing home organizations in different regions in the Netherlands were recruited for participation (Figure 1). The number of residents per unit ranged from 3 to 32. Table 2 shows the baseline characteristics of residents and staff. Staff baseline characteristics were taken from a survey completed by 319 staff members (84.8%). The intervention and control groups differed in terms of the proportions of staff in permanent positions. There were no other statistically significant differences at baseline between the intervention and control groups.

**Table 2:** Baseline Characteristics.

Nursing Homes		
	Intervention Group (n = 5)	Usual Care Group (n = 6)
Number of nursing homes	5	6
Number of units	13	21
Number of residents per unit (mean and SD)	13^.^5 (8^.^2)	8^.^80 (4^.^47)
Number of staff members per unit (mean and SD)	14^.^0 (7^.^4)	9^.^28 (6^.^61)
Number of staff per resident (mean and SD)	0^.^17 (0^.^04)	0^.^18 (0^.^01)
**Residents**		
	**Intervention Group (n = 154)**	**Usual Care Group (n = 164)**
Mean age in years (SD)	84^.^8 (6^.^0)	84^.^59 (6^.^6)
Women	118 (76^.^6%)	121 (73^.^8%)
**Staff**		
	**Intervention Group (n = 141)**	**Usual Care Group (n = 178)**
Mean age in years (SD)	43^.^6 (10^.^4)	42^.^6 (11^.^3)
Women	139 (98^.^6%)	175 (98^.^3%)
Born in the Netherlands	129 (91^.^5%)	160 (89^.^9%)
Years working in the current position (mean and SD)	10^.^3 (8^.^3)	10^.^0 (8^.^6)
Years working in the organization (mean and SD)	12^.^8 (8^.^1)	10^.^1 (7^.^9)
Permanent employment contract	139 (98^.^5%)	163 (91^.^6%)
Number of hours a week by contract (mean and SD)	23^.^7 (6^.^7)	22^.^6 (7^.^2)
Previous interest in or experience with person-centered care	79 (56^.^0%)	99 (55^.^6%)

### Costs

**Figure 2:**
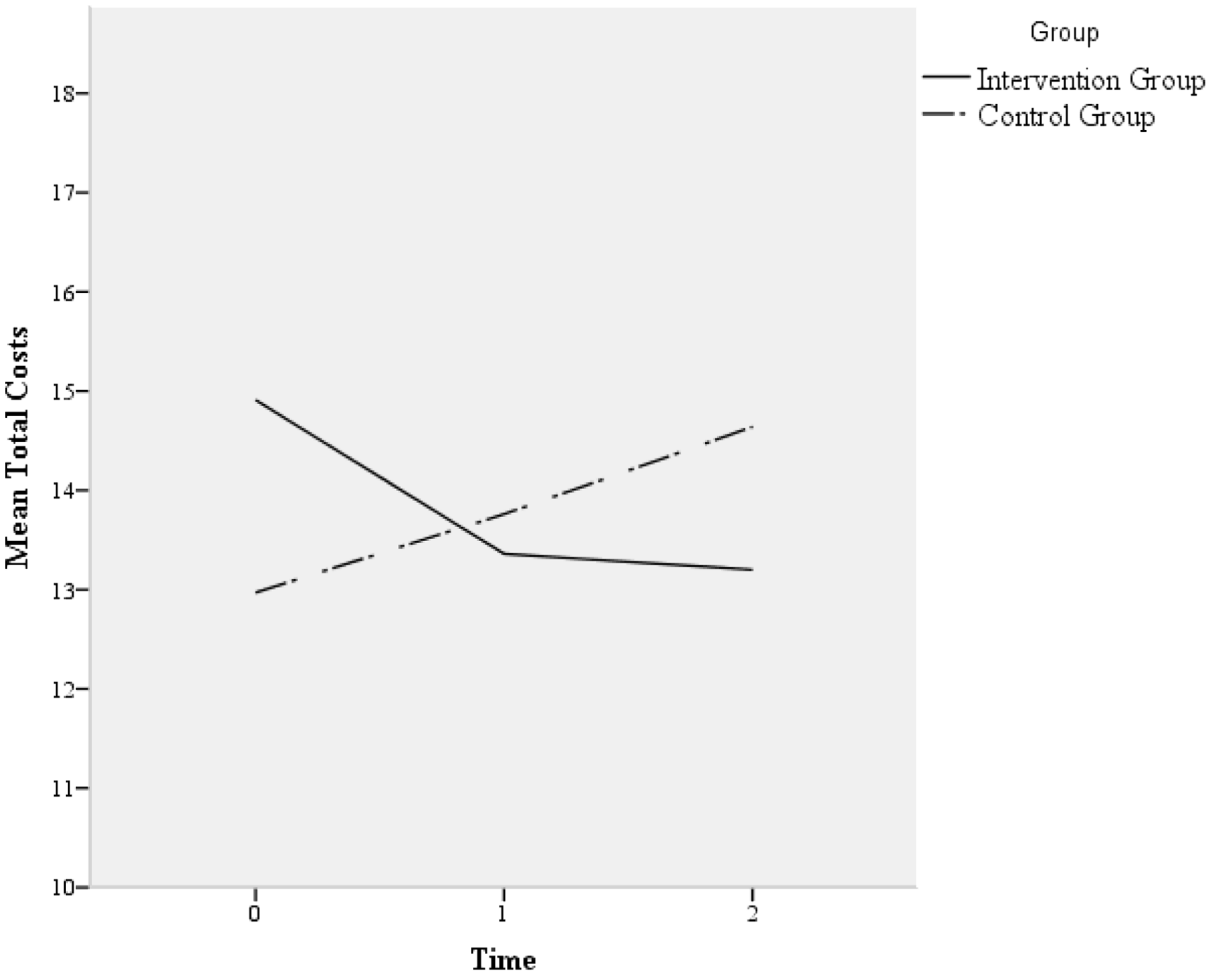
Mean total costs per resident per day in US dollars.

Analyses showed no effect of the intervention on total costs (p = 0^.^60). The total costs included residents’ healthcare consumption and drug use, staff absenteeism, and the costs of the DCM intervention. Figure 2 shows the mean total costs per resident per day. There were no differences between the intervention and control groups for the total residents’ costs (healthcare consumption and drug use). On the staff level, there was no significant difference between the intervention and control group for costs associated with absenteeism. In both groups, the number of falls, costs of care provided by the elderly-care physicians and nurse practitioners, increased over time (p<0^.^02), but no significant interaction between group and time was found.

Compared to the control group, the intervention group showed a decrease in costs associated with outpatient hospital appointments over time (p = 0^.^05). The use of psychotropic drugs decreased over time in both groups (p = 0^.^01 for time effect). We found a significant interaction for the psychotropic drug use. However, the interaction pattern did not clearly favour either the intervention group or the control group.

The mean DCM implementation costs per resident per day were US $0.63 (SD $0.23) (see Table 3). The findings just outlined were not affected by the exclusion of the DCM implementation costs from the model. Table 4 shows the means and SEs for the intervention and control groups for all outcome measures.

**Table 3:** Intervention costs.

	Hours invested per unit (mean and SD)	Mean costs hours invested (hourly wages $27.68)	Training costs
**DCM basic training**	32 hours (0^.^00)	$885^.^76	$979^.^99
**DCM advanced training**	32 hours (0^.^00)	$885^.^76	$1371^.^98
**Mapping exercise**	6 hours (0^.^00)	$166^.^08	
**Inter-rater reliability test**	1^.^5 hour (0^.^00)	$41^.^52	
**Observation**	20^.^85 hours (11^.^20)	$577^.^13	
**Preparing DCM reports**	28.43 hours (15^.^03)	$786^.^94	
**Feedback sessions**	6^.^89 hours (4^.^14)	$190^.^72	
**Total intervention costs per unit (mean and SD)**	$2856^.^81 ($365^.^86)		
**Costs per resident per day (mean and SD)**	$0^.^63 ($0^.^23)		

**Table 4:** Results (Costs) for Residents and Staff.

	Intervention Group			Usual Care Group					
Residents	T0 n = 154	T1 n = 144	T2 n = 137	T0 n = 164	T1 n = 166	T2 n = 156	Baseline ICC	Signifi-cance	
**Mean annual number and SE of falls per resident**									
Falls	2^.^78 (0^.^63)	3^.^13 (0^.^40)	3^.^33 (0^.^39)	1^.^41 (0^.^64)	1^.^48 (0^.^41)	1^.^81 (0^.^39)	0.03	p_g = _0^.^11 p_t = _0^.^023 p_gt = _0^.^799	
**Mean costs of healthcare consumption per resident per day in US dollars and SE**									
Elderly-care physician and nurse practitioner	2^.^52 (0^.^34)	2^.^60 (0^.^45)	2^.^84 (0^.^40)	2^.^83 (0^.^32)	4^.^05 (0^.^41)	3^.^73 (0^.^36)	0.08	p_g = _0^.^07 p_t = _0^.^011 p_gt = _0^.^067	
Psychologist	1^.^03 (0^.^17)	0^.^93 (0^.^15)	0^.^74 (0^.^14)	0^.^88 (0^.^18)	1^.^25 (0^.^15)	1^.^23 (0^.^15)	0.00	p_g = _0^.^12 p_t = _0^.^636 p_gt = _0^.^126	
Paramedical professionals	0^.^74 (0^.^12)	0^.^68 (0^.^13)	0^.^72 (0^.^13)	0.55 (0^.^11)	0^.^75 (0^.^12)	0^.^76 (0^.^16)	0.06	p_g = _0^.^84 p_t = _0^.^506 p_gt = _0^.^189	
Outpatient Hospital Contacts	1^.^00 (0^.^14)	0^.^87 (0^.^12)	0^.^65 (0^.^16)	0^.^91 (0^.^15)	0^.^71 (0^.^12)	1^.^04 (0^.^14)	0.18	p_g = _0^.^78 p_t = _0^.^313 p_gt = _0^.^050	
Hospital Admissions	0^.^70 (0^.^26)	0^.^53 (0^.^28)	0^.^86 (0^.^30)	0^.^45 (0^.^24)	0^.^54 (0^.^25)	0^.^11 (0^.^27)	0.01	p_g = _0^.^23 p_t = _0^.^939 p_gt = _0^.^322	
Total healthcare consumption	3^.^28 (0^.^42)	2^.^92 (0^.^39)	3^.^50 (0^.^46)	3^.^10 (0^.^39)	3^.^19 (0^.^36)	3^.^23 (0^.^43)	0.08	p_g = _0^.^90 p_t = _0^.^545 p_gt = _0^.^541	
Psychotropic drug use	0^.^20 (0^.^07)	0^.^32 (0^.^08)	0^.^13 (0^.^06)	0^.^36 (0^.^07)	0^.^28 (0^.^08)	0^.^25 (0^.^06)	0.03	p_g = _0^.^40 p_t = _0^.^011 p_gt = _0^.^032	
Total resident-based costs: health care consumption and drug use	4^.^16 (0^.^62)	3^.^83 (0^.^66)	4^.^25 (0^.^59)	4^.^02 (0^.^62)	3^.^83 (0^.^66)	4^.^40 (0^.^57)	0.04	p_g = _0^.^59 p_t = _0^.^514 p_gt = _0^.^193	
	**Intervention Group**			**Usual Care Group**					
**Staff**	**T0 n = 178**	**T1 n = 183**	**T2 n = 184**	**T0 n = 198**	**T1 n = 199**	**T2 n = 188**		**Signifi-cance**	
**Mean costs per unit per day in US dollars and SE**									
Absenteeism	9^.^55 (2^.^23)	8^.^79 (1^.^59)	8^.^57 (1^.^69)	7^.^25 (2^.^78)	7^.^53 (2^.^04)	9^.^92 (2^.^17)	0.05	p_g = _0^.^42 p_t = _0^.^793 p_gt = _0^.^249	
	**Intervention Group**			**Usual Care Group**					
**Total costs including DCM intervention**	**T0**	**T1**	**T2**	**T0**	**T1**	**T2**		**Signifi-cance**	
**Mean costs per resident per day in US dollars and SE**									
Total costs healthcare consumption, drug use, absenteeism, and intervention costs	14^.^91 (2^.^29)	13^.^36 (1^.^65)	13^.^20 (1^.^92)	12^.^97 (3^.^12)	13^.^76 (2^.^11)	14^.^64 (2^.^44)	0.02	p_g = _0^.^93 p_t = _0^.^991 p_gt = _0^.^604	

DCM = dementia-care mapping.

SE = standard error.

*P*_g_ = main effect of the groups.

*P*_t_ = main effect of time.

*P*_gt_ = interaction between group and time.

## Discussion

Overall, DCM turned out to be a cost-neutral intervention, sustaining affordability of institutionalized dementia care. The intervention group did show lower costs associated with outpatient hospital appointments than the control group during the evaluation period. The relationship between this cost saving effect and the DCM intervention is not clear. The effects on costs did not change when the DCM implementation costs were eliminated from the model, which means that these costs are negligible compared to the costs associated with daily care.

The average number of falls corresponds with the numbers found in previous studies in Dutch nursing homes [19]. In contrast to Chenoweth and colleagues’ study [20], we found no reduction in falls. Chenoweth et al. calculated the proportion of residents who did fall, whereas in this study we collected the registered number of falls at the unit level. This was done for practical reasons concerning the registration of falls in the nursing home records. There is no reason to believe that this difference in approach influenced the results. Importantly, in long-term care facilities like nursing homes, it seems to be difficult to reduce the number of falls, even when, unlike DCM, an intervention focuses on preventing falls. [21].

The use of psychotropic drugs decreased in both groups over time. Chenoweth and colleagues [20] found no significant effect of DCM on drug use. Despite the reluctance of physicians to change their pharmaceutical prescribing habits [22], the decrease in psychotropic drug use can be explained as a result of a steady change in the policy of elderly-care physicians to decrease the prescription of inappropriate psychotropic drugs.

The main strengths of this study are the large sample size, cluster randomization, and the relatively long study period of 18 months. We cluster-randomized the units after recruiting the residents and seeking informed consent. This way, we controlled for selection bias in the control- and intervention groups. We used the minimization method in randomization to optimize distribution of baseline characteristics across the intervention and control groups.

This study has several limitations. First, we were unable to blind participating staff to the intervention, given the necessity of staff training in DCM. Second, we cannot guarantee that the units were representative of Dutch nursing homes – they agreed to participate in this study because they were at least interested in PCC and DCM. Furthermore, the nursing home data and hospital care appointments were extracted from residents’ medical files. There is variation in the way health care professionals register their contacts with the residents. Some nursing homes had structured electronic files, while others had paper files that made it difficult to extract all the necessary information. In both cases, there may be some under-registration. Particularly the drug files for the residents who had died or relocated were often unavailable. However, there is no reason to believe that the rates of under-registration differ between the intervention and control groups. Finally, we did not measure the time nurses spent on different tasks or residents. Because the nurses work a fixed number by contract, it was difficult to recover the data for differences in time spent on the actual care delivery. If anything, we would expect that the DCM intervention increased the proportion of time spent on tailored care.

We find that DCM is a cost-neutral intervention for nursing home residents with dementia that has an advantage over usual care when it comes to the costs of outpatient hospital appointments.Since DCM has shown positive effects on resident outcome measures such as depression, agitation and quality of life [20], [23], considerations other than costs may determine whether or not a nursing home will adopt this method.

## Supporting Information

Checklist S1 **CONSORT Checklist.**
(DOC)

Protocol S1 **Trial Protocol.**
(PDF)

Research Proposal S1 **Research Proposal.**
(PDF)
